# 2687. Tetracycline Resistance in *Neisseria gonorrhoeae* Isolates Among Transgender Women and Men Who Have Sex with Men — eGISP and SURRG, 2018–2021

**DOI:** 10.1093/ofid/ofad500.2298

**Published:** 2023-11-27

**Authors:** Sarah Wondmeneh, Emily Learner, Matthew W Schmerer, Alesia Harvey, Kerry Mauk, Laura A Quilter, Sancta St. Cyr

**Affiliations:** Centers for Disease Control and Prevention, Atlanta, Georgia; Centers for Disease Control and Prevention, Atlanta, Georgia; Centers for Disease Control and Prevention, Atlanta, Georgia; Centers for Disease Control and Prevention, Atlanta, Georgia; Centers for Disease Control and Prevention, Atlanta, Georgia; Centers for Disease Control and Prevention, Atlanta, Georgia; Centers for Disease Control and Prevention, Atlanta, Georgia

## Abstract

**Background:**

Doxycycline, a tetracycline analog, is being considered for use as post-exposure prophylaxis (PEP) to reduce bacterial sexually transmitted infections (STIs) among gay, bisexual, and other men who have sex with men (MSM) and transgender women. Pre-existing tetracycline resistance may contribute to the varied effectiveness of doxycycline in preventing *Neisseria gonorrhoeae* (GC). We examined trends in tetracycline resistance (TetR) and tetM plasmid-mediated high-level tetracycline resistance (TetHLR) among GC isolates collected from MSM and transgender women through national sentinel surveillance.

**Methods:**

Genital (urethral, urine, vaginal or endocervical), pharyngeal and rectal GC isolates were collected from MSM and transgender women during 2018–2021 at 12 Enhanced Gonococcal Isolate Surveillance Project (eGISP) and 8 Strengthening the U.S. Response to Resistant Gonorrhea (SURRG) sites. We determined overall prevalence of TetR and TetHLR (minimum inhibitory concentration, MIC ≥2 µg/mL and MIC ≥16 µg/mL, respectively) among MSM and transgender women by anatomic site of infection and described annual trends among MSM.

**Results:**

Among 10,853 isolates collected from 8,667 MSM, overall TetR prevalence was 31% (genital: 31%, pharyngeal: 32%, rectal: 29%), and annual TetR prevalence decreased during 2018–2021 (Table 1). Overall TetHLR prevalence among MSM was 13% (genital: 13%, pharyngeal: 12%, rectal: 12%) and annual TetHLR prevalence for genital and pharyngeal infections increased during 2018–2021. Among 108 isolates collected from 89 transgender women, overall TetR prevalence was 23% (genital: 19%, pharyngeal: 23%, rectal: 25%) and TetHLR prevalence was 9% (genital: 0%, pharyngeal: 10%, rectal: 12%), though numbers were small.

**Figure:**
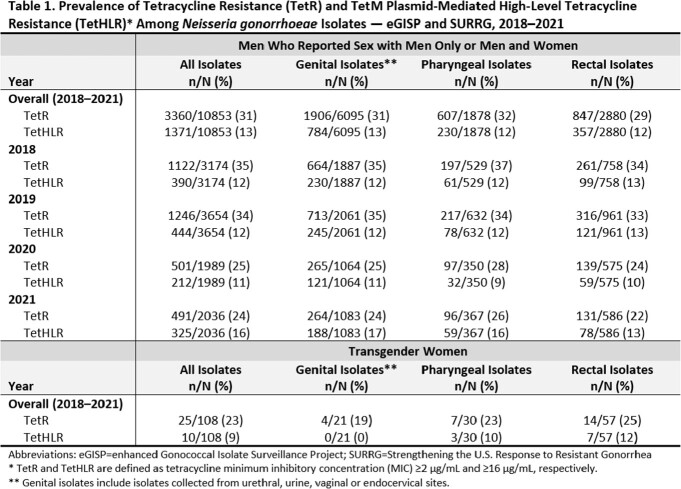

**Conclusion:**

Approximately 1/3 of GC isolates collected from MSM and 1/4 of GC isolates collected from transgender women were tetracycline resistant. Prevalence of tetracycline resistance was similar across anatomic sites. Providers should continue to follow recommended screening and treatment guidelines for gonorrhea among persons using doxycycline as STI PEP. Additionally, given high levels of TetHLR, enhanced tetracycline resistance surveillance is needed with implementation of doxycycline as STI PEP.

**Disclosures:**

**All Authors**: No reported disclosures

